# Management of obstructive jaundice induced by a retained bullet in the common hepatic duct: A case report

**DOI:** 10.5339/qmj.2025.62

**Published:** 2025-06-11

**Authors:** José A. Rodriguez Zamboni, Martin J. Drago, Lucila Fregonese, Ricardo Reverendo, Luis E. Sarotto

**Affiliations:** 1 General Surgery Department, Hospital Central de Emergencia y Alta Complejidad de Pilar, Buenos Aires, Argentina *Email: agustinzamboni@gmail.com

**Keywords:** Obstructive jaundice, common hepatic duct, foreign body, bullet injury, biliary surgery

## Abstract

**Background:**

Traumatic injuries to the extrahepatic bile ducts are rare, with an incidence of 0.4%–0.6% in cholecystectomy procedures among adults, particularly following the introduction of laparoscopic cholecystectomy. Among these, the presence of foreign bodies within the biliary tree is exceptionally rare, with obstructive jaundice caused by a bullet lodged in the common hepatic duct being particularly uncommon. This case report aims to share the diagnostic process and the challenges in managing such a rare condition.

**Case Presentation:**

A 41-year-old female with a 13-year history of an accidental gunshot wound, which required an emergency laparotomy, presented to our hospital with symptoms of cholangitis. Endoscopic retrograde cholangiopancreatography was performed, revealing a bullet that caused significant dilation of the proximal bile duct. Exploratory laparoscopy, cholecystectomy, and intraoperative cholangiography confirmed the presence of the bullet. The procedure included a choledochotomy and removal of the bullet, followed by primary closure of the common bile duct using interrupted 4-0 Prolene sutures. The patient was discharged on the 11th postoperative day, and follow-up revealed complete resolution of symptoms and normal liver function.

**Discussion:**

This case underscores the rarity and complexity of managing extrahepatic bile duct injuries caused by foreign bodies. The delayed presentation of symptoms and the unique diagnostic challenges highlight the necessity for meticulous imaging. The successful surgical intervention in this case highlights the importance of individualized treatment strategies implemented by a multidisciplinary team.

**Conclusion:**

Managing traumatic injuries to the extrahepatic bile ducts requires careful consideration due to their rarity and complexity. The challenges in diagnosis and treatment underscore the necessity of a multidisciplinary approach.

## Background

Foreign bodies that remain embedded in the human body for extended periods without causing symptoms are rare but not unheard of. In some cases, these objects are unknowingly retained, or their removal is deemed too risky due to potential complications. While asymptomatic retention can persist for years, delayed-onset complications, such as infection, obstruction, or erosion into adjacent structures, present significant diagnostic and therapeutic challenges.^1^

Traumatic injuries to the extrahepatic bile ducts are rare, with an incidence of 0.4%–0.6% in adults following the introduction of laparoscopic cholecystectomy.^2,3^ These injuries are associated with high morbidity and mortality rates.^4–6^ Accurate diagnosis is crucial for optimizing treatment and improving outcomes.^7–9^

Foreign bodies within the biliary tree, as a cause of obstructive jaundice, are particularly uncommon.^7^ Obstructive jaundice caused by a bullet lodged in the common hepatic duct is an exceedingly rare event. The presence of a foreign body, such as a bullet, in the biliary system presents unique diagnostic and therapeutic challenges. Management of such cases is particularly complex, and the literature on this topic is limited.

Our healthcare facility, located within a tertiary care center, is equipped with the resources and expertise to manage such rare and complex cases. Therefore, we report a case of late-onset obstruction of the common hepatic duct due to an unusual foreign body.

Written consent was obtained from the patient to use medical records and images in the publication of this manuscript.

## Case Presentation

A 41-year-old female was referred to our hospital by her local primary care physician due to progressive jaundice and abdominal pain. She reported to the General Surgery Department with a chief complaint of a 7-day history of right upper quadrant abdominal pain, associated with nausea progressing to vomiting, jaundice, choluria, and acholia.

Her medical history included an accidental gunshot injury 13 years ago, which required emergency exploratory laparotomy at another hospital at that time. Operative notes from that procedure indicated no solid organ injury or hollow viscus perforation, and hemostasis was achieved; however, there was no record of the abdominal bullet being retrieved.

On presentation, her vital signs were stable, and she was afebrile. Physical examination revealed generalized jaundice, tenderness in the epigastrium and right upper quadrant, and a midline abdominal scar consistent with her surgical history. Laboratory investigations showed leukocytosis (11.7 × 10^9^/L) and elevated liver enzyme levels: AST 237 IU/L, ALT 268 IU/L, GGT 160 U/L, and amylase 27 U/L. The total bilirubin was markedly elevated at 13 mg/dL, predominantly direct bilirubin (normal up to 2.2 mg/dL). Alkaline phosphatase was significantly elevated at 1,815 IU/L. Inflammatory markers included a C-reactive protein level of 317.38 mg/L and an erythrocyte sedimentation rate exceeding 120 mm.

**Figure 1:**
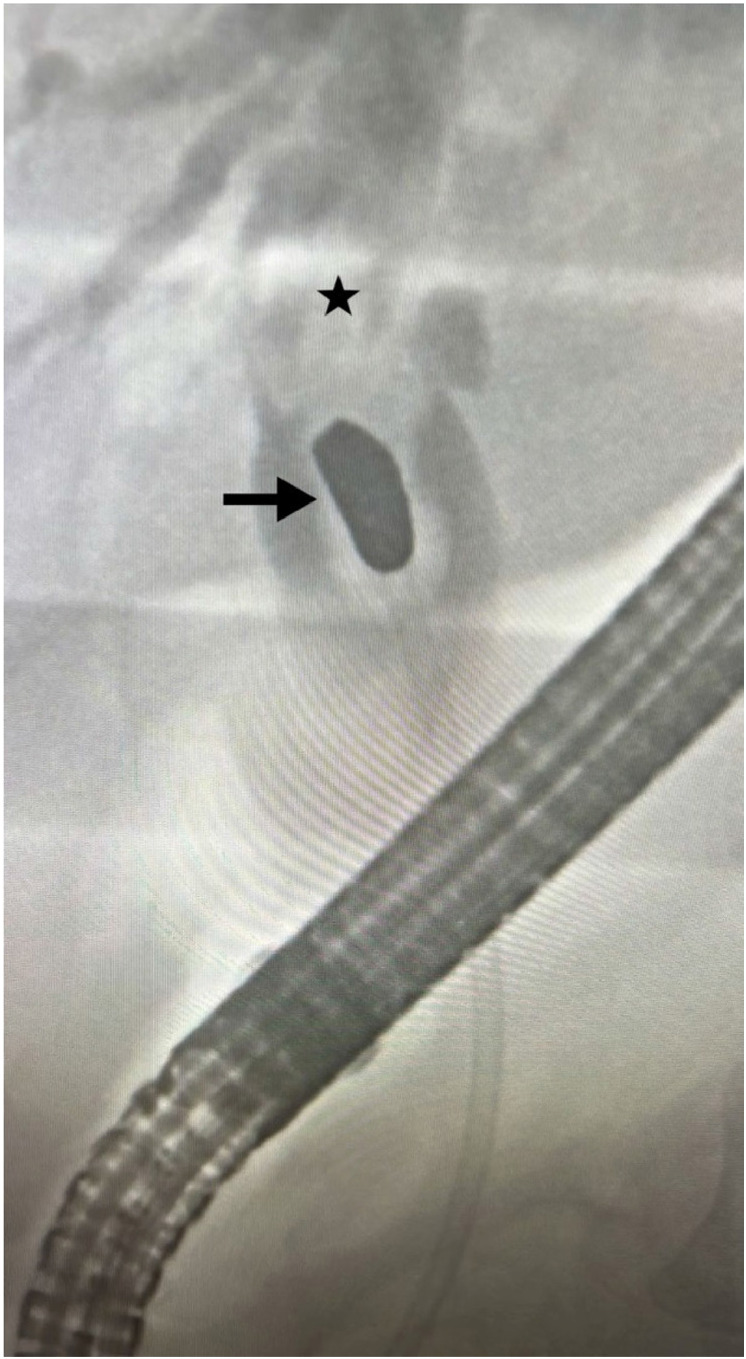
ERCP revealing a bullet-shaped foreign body (arrow) in the CBD (star), with significant proximal dilation observed.

An abdominal ultrasound revealed 3 mm dilation of the intrahepatic bile ducts and 12 mm dilation of the extrahepatic bile duct, along with a 17 mm hyperechogenic image in the proximal common bile duct (CBD). Cholangitis was diagnosed, and an urgent endoscopic retrograde cholangiopancreatography (ERCP) was performed. This procedure revealed a distal CBD of normal caliber, but significant proximal dilation up to 18 mm was observed, initially suggestive of a biliary stone. After complete opacification, a foreign body with blunt edges, approximately 3 cm in size and resembling a bullet, was identified in the proximal CBD (Figure 1). Following papillotomy, migration of the foreign body was unsuccessful. Biliary drainage was then attempted using an 8.5 Fr by 10 cm stent, which raised suspicion of spontaneous migration of the bullet into the CBD. Consequently, surgical exploration was deemed necessary. Exploratory laparoscopy revealed a distended gallbladder with edematous walls and multiple stones, along with extensive adhesions of the greater omentum to the midline and anterior hepatic surface.

**Figure 2:**
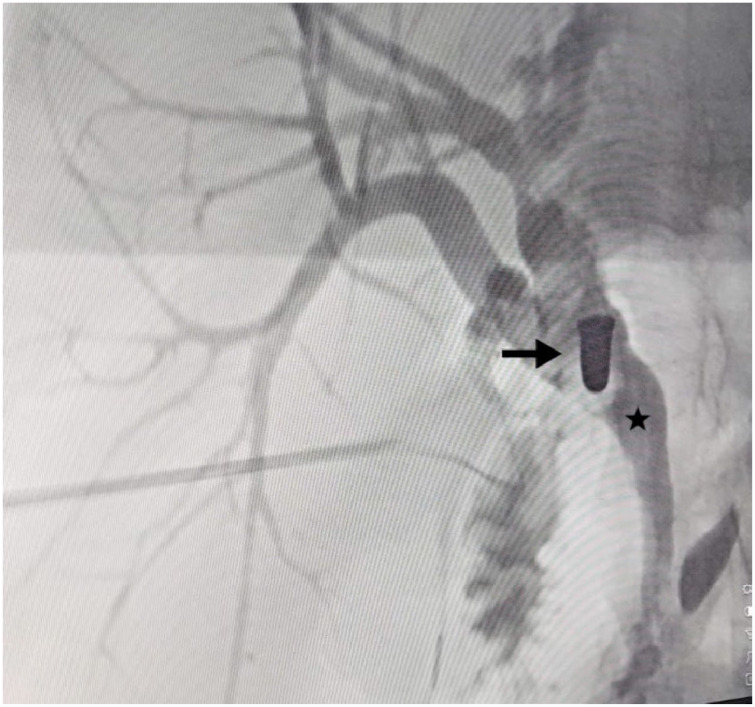
An IOC revealed a radiopaque, bullet-shaped object (arrow) located in the middle third of the common hepatic duct (star), with proximal dilation.

**Figure 3:**
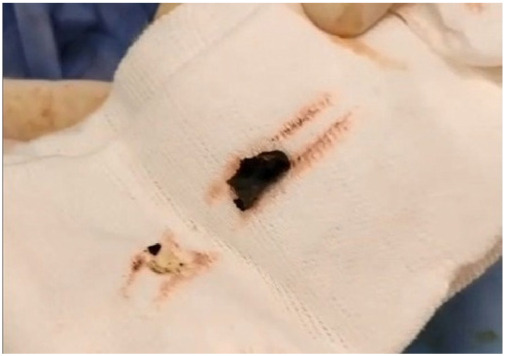
Bullet removed from the CBD using transcystic forceps.

**Figure 4:**
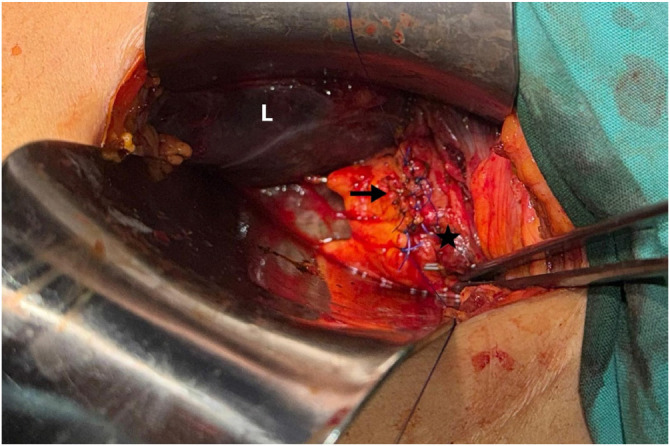
Closure of the CBD (star) with interrupted 4-0 Prolene sutures (arrow). L: liver.

After the dissection of the Calot’s triangle, the surgeon determined that Strasberg’s critical view of safety had been achieved. A cholangiography catheter was inserted into the cystic duct, and intraoperative cholangiography (IOC) was performed, revealing the CBD dilated to 12 mm, with a radiopaque bullet shape object located in the middle third, and the biliary stent appropriately inserted (Figure 2). A cholecystectomy was then performed from the neck to the fundus, and the gallbladder was removed through the epigastric trocar. A 1 cm choledochotomy was performed at the site of the cystic duct insertion. A Dormia basket was inserted through the bile duct, but extraction of the foreign body was unsuccessful. Consequently, the procedure was converted to open surgery via a minimal subcostal incision. The bullet was successfully removed from the CBD using transcystic forceps (Figure 3). Primary closure of the bile duct was achieved with interrupted 4-0 Prolene sutures (Figure 4). Bile leak testing was satisfactory.

Serum bilirubin and alkaline phosphatase levels returned to normal in the early postoperative period. The patient was discharged on the 11th postoperative day without further complications. At the 1-month follow-up, the patient remained asymptomatic, with no evidence of liver function abnormalities on laboratory tests.

## Discussion

Extrahepatic biliary tree injury occurs in approximately 0.1% of adults and 0.009% of pediatric trauma cases.^10^ Isolated extrahepatic biliary tree injury is extremely rare, occurring in only 2–3% of such cases.^11,12^ Furthermore, a late presentation with bile duct obstruction secondary to previous abdominal trauma is even more uncommon.^13^

Previous studies have reported cholestatic syndrome caused by a bullet from a prior event. One case has been reported in the pediatric age group,^14^ while others, similar to our case, have been reported in the adult population.^13,15–17^ It is notable that a lodged bullet may remain asymptomatic for an extended period, with complications such as obstructive jaundice potentially arising many years postinjury, as demonstrated in this case. Patients may also present intermittently with symptoms resembling biliary colic, which may be associated with the migration of a foreign body through the bile ducts.^17^ Cholangitis can also occur; the presence of a foreign body increases the risk of infection and may also lead to pancreatitis.^18^ The onset of symptoms is not precise and may take years for a cholestatic presentation to develop. Some authors have suggested that the delayed presentation is likely due to the migration of the bullet from its original location to the biliary tree.^17^

Migration is rare, occurring in less than 5% of cases and often resulting in complications involving surrounding tissues and organs.^19^ Bullets retained in soft tissue are typically encapsulated by fibrous scar tissue, which reduces the likelihood of migration.^20^ In contrast, retained missiles or fragments may erode into open tissue spaces, potentially years after the initial injury.^21^

Diagnosis may require imaging to visualize the site and potential cause of the obstruction. Initially, simple ultrasonography can be used to assess bile duct dilation and characterize obstructions and is also recommended for detecting fluid collections in the gallbladder fossa or ductal dilation.^22^ Additionally, abdominal radiography can help identify the presence of metallic objects, although it may not pinpoint the exact location of the obstruction.^7,13^ Computed tomography (CT) offers several advantages over ultrasound, including a sensitivity greater than 83% and a specificity of around 83% for diagnosing acute cholangitis, as well as the ability to identify metallic objects.^22,23^ Additionally, CT is valuable for detecting complications such as hepatic abscesses and portal thrombosis and for ruling out other potential causes of abdominal pain.^24^ Other studies have reported that the diagnosis was confirmed through a perioperative cholangiogram during surgery.^15^ If obstruction of a biliary duct due to a foreign body, especially a metallic object or bullet, is suspected, magnetic resonance cholangiopancreatography (MRCP) should not be performed. MRCP does not offer additional benefits for patient management and may interfere with the bullet’s position in the bile duct, potentially causing iatrogenic injury. Tamasauskas et al. report a case of bile duct obstruction caused by a projectile, in which MRCP was performed, leading to significant iatrogenic injury of the bile duct without altering the management of the case.^25^

Once the diagnosis is established, ERCP should be performed. The primary goal is a diagnostic approach that enables the identification of the object’s position within the bile duct and, if possible, its removal.^16^ However, if endoscopy fails to extract the foreign body from the bile duct, surgical intervention should be promptly performed. In our case, an ERCP was performed but failed to extract the foreign body, requiring CBD drainage with a biliary stent due to cholangitis. Subsequently, a laparoscopy was scheduled, followed by cholecystectomy and IOC to confirm the suspected diagnosis and identify the location of the foreign body. We then conducted a choledochotomy and extracted the bullet, with a primary closure of the CBD using interrupted 4-0 Prolene sutures, similar to what is mentioned in previous studies.^13,15–17^

## Conclusion

Managing traumatic injuries to the extrahepatic bile ducts, particularly when caused by a foreign body such as a bullet, presents both rarity and complexity. The delayed onset of symptoms and the challenges in diagnosis and treatment underscore the need for careful imaging and a multidisciplinary approach. In this case, surgical intervention was necessary to effectively manage the obstruction, and the patient had an uneventful recovery with resolution of symptoms. This highlights the importance of individualized treatment strategies for such rare and challenging cases, which must be conducted by specialized and multidisciplinary teams.

## Ethical approval, consent to participate, and IRB statement

Written consent was obtained from the patient to use medical records and images in the publication of this manuscript.

## Conflicts of interest

The authors declare that they have no competing interests.

## References

[1] David Richardson J, Franklin GA, Lukan JK, Carrillo EH, Spain DA, Miller FB (2000;). Evolution in the management of hepatic trauma: a 25-year perspective. Ann Surg.

[2] Kapoor VK, BjörnTörnqvist (2020). Epidemiology of bile duct injury. In: Post-cholecystectomy bile duct injury.

[3] Alexander HC, Bartlett AS, Wells CI, Hannam JA, Moore MR, Poole GH (2018;). Reporting of complications after laparoscopic cholecystectomy: a systematic review. HPB (Oxford).

[4] Booij KAC, de Reuver PR, van Dieren S, van Delden OM, Rauws EA, Busch OR (2018;). Long-term impact of bile duct injury on morbidity, mortality, quality of life, and work related limitations. Ann Surg.

[5] Fong ZV, Pitt HA, Strasberg SM, Loehrer AP, Sicklick JK, Talamini MA (2018;). Diminished survival in patients with bile leak and ductal injury: management strategy and outcomes. J Am Coll Surg.

[6] Halbert C, Altieri MS, Yang J, Meng Z, Chen H, Talamini M (2016;). Long-term outcomes of patients with common bile duct injury following laparoscopic cholecystectomy. Surg Endosc.

[7] Kamona A, Mansour A, Qandeel M, Al-Eshaiker M (2005;). Biliary obstruction secondary to combat-related foreign bodies: report of two cases. Abdom Imaging.

[8] Fletcher R, Cortina CS, Kornfield H, Varelas A, Li R, Veenstra B (2019;). Bile duct injuries: a contemporary survey of surgeon attitudes and experiences. Surg Endosc.

[9] Mercado MA, Dominguez I (2011;). Classification and management of bile duct injuries. World J Gastrointest Surg.

[10] Hecker A, Hecker M, Riedel JG, Hecker B, Doppstadt C, Weigand MA (2020;). Neue WSES-AAST-Leitlinie zum Trauma des Duodenums, des Pankreas und der extrahepatischen Gallengänge – Zusammenfassung und Kommentar [New WSES-AAST guideline on duodeno-pancreatic and extrahepatic biliary tree trauma-summary and comments]. Chirurg.

[11] Soukup ES, Russell KW, Metzger R, Scaife ER, Barnhart DC, Rollins MD (2014;). Treatment and outcome of traumatic biliary injuries in children. J Pediatr Surg.

[12] Thomson BN, Nardino B, Gumm K, Robertson AJ, Knowles BP, Collier NA (2012;). Management of blunt and penetrating biliary tract trauma. J Trauma Acute Care Surg.

[13] De Macedo FPPC, Maués CAD, Mendes Filho O, da Costa KG, Rodriguez JER, Csasznik I (2019;). Late cholestatic syndrome due to previous perforating trauma: case report. Int J Surg Case Rep.

[14] Rescorla FJ, Schlatter M, Hawes RH, Grosfeld JL (1996;). Delayed presentation of a penetrating biliary tract injury in a child. J Trauma.

[15] Maheshwari M, Chawla A, Dalvi A, Thapar P, Raut A (2003;). Bullet in the common hepatic duct: a cause of obstructive jaundice. Clin Radiol.

[16] Calderon AJ, Irabien M (2021;). Endoscopic removal by ERCP of a foreign body (bullet) in the common bile duct. Gastrointest Endosc.

[17] Hussain SM, Zulqurnain S, Saleem O (2007;). Delayed obstructive jaundice secondary to bullet in common hepatic duct. J Coll Physicians Surg Pak.

[18] Kim KH, Woo EY, Rosato EF, Kochman ML (2004;). Pancreatic foreign body: ingested toothpick as a cause of pancreatitis and hemorrhage. Gastrointest Endosc.

[19] Rapp LG, Arce CA, McKenzie R, Darmody WR, Guyot DR, Michael DB (1999;). Incidence of intracranial bullet fragment migration. Neurol Res.

[20] Nickel WN, Steelman TJ, Sabath ZR, Potter BK (2018;). Extra-articular retained missiles; Is surveillance of lead levels needed?. Mil Med.

[21] Marantidis J, Biggs G (2019;). Migrated bullet in the bladder presenting 18 years after a gunshot wound. Urol Case Rep.

[22] Moghul F, Kashyap S (2023). Bile duct injury. In: StatPearls.

[23] Kim SW, Shin HC, Kim HC, Hong MJ, Kim IY (2012;). Diagnostic performance of multidetector CT for acute cholangitis: evaluation of a CT scoring method. Br J Radiol.

[24] Sokal A, Sauvanet A, Fantin B, de Lastours V (2019;). Acute cholangitis: diagnosis and management. J Visc Surg.

[25] Tamasauskas I, Roberto J, Carlotto M, Apodaca FR, Goldenberg A, Lobo EJ (2016;). Biliary tract obstruction secondary to a foreign body: chambered projectile in common hepatic duct. Relatos Caso do CBC.

